# Development of sustainable carbon fiber composites using dual dynamic epoxy vitrimers: a synergy of stiffness, flexibility, and recyclability

**DOI:** 10.1039/d5na00624d

**Published:** 2025-08-04

**Authors:** Harsh Sharma, Viranchika Bijalwan, Sravendra Rana

**Affiliations:** a School of Advanced Engineering, UPES, Energy Acres Bidholi Dehradun Uttarakhand 248007 India srana@ddn.upes.ac.in

## Abstract

The increasing demand of carbon fiber-reinforced polymer (CFRP) composites in sectors such as aerospace and wind energy underscores the need for sustainable end-of-life solutions. In this work, a dual dynamic bio-based epoxy consisting of covalent adaptable networks (vitrimers) was developed by blending diglycidyl ether of bisphenol-A (DGEBA) with acrylated epoxidized soybean oil (AESO) in varying ratios, cured with 2,2′-dithiodibenzoic acid (DTBA), and transesterification catalyzed by tin(ii) 2-ethylhexanoate (Sn(Oct)_2_). Among the formulations, D70B30 and D50B50 vitrimers exhibited a synergistic balance between mechanical stiffness and flexibility, owing to the aromatic rigidity of DGEBA and the aliphatic softness of AESO. Both vitrimers demonstrated excellent thermal stability (*T*_d5%_ = 342 °C and 325 °C, respectively), high gel content (>99%), and outstanding self-healing efficiencies (∼92% for D70B30, ∼90% for D50B50) due to dynamic transesterification and disulfide bond exchange mechanisms. They also exhibited rapid stress relaxation and efficient degradability, confirming their vitrimeric behavior. These optimized matrices were used to fabricate CFRP laminates *via* vacuum-assisted resin infusion molding (VARIM), resulting in composites with remarkable mechanical performance. D70B30-CF showed superior tensile strength (281 MPa) and flexural strength (600 MPa), while D50B50-CF exhibited a more rigid response with higher flexural modulus (58.7 GPa). Additionally, the vitrimer matrix allowed for efficient chemical recycling in EG/DMF at 70 °C, enabling complete matrix dissolution and full recovery of undamaged carbon fibers within 4 hours. Structural and morphological integrity of the recycled fibers was confirmed through FTIR, XRD, and SEM analysis. This study presents a viable strategy for developing high-performance, reprocessable, and recyclable CFRPs using sustainable covalent adaptable networks.

## Abstract 2

Dual dynamic epoxy vitrimers enable self-healing, reprocessing, and recycling of carbon fiber composites with balanced stiffness, flexibility, and mechanical performance are reported.

## Graphical abstract

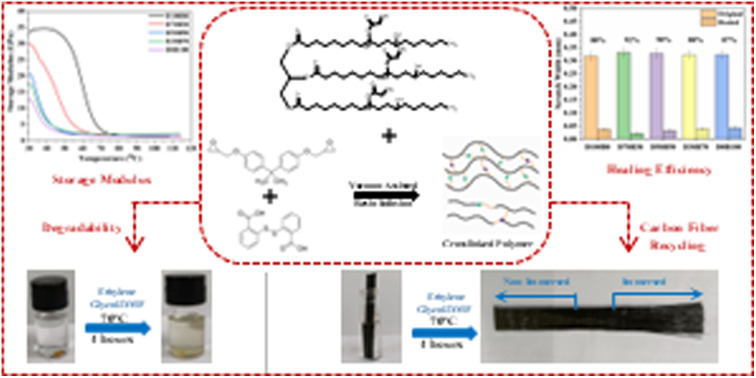

## Introduction

Owing to their remarkable mechanical properties, high strength-to-weight ratios, thermal stability, and toughness, carbon fiber-reinforced polymer (CFRP) composites have attracted the interest of numerous industries.^1,2^ These materials are utilized in high-performance applications such as aerospace, automotive, wind turbines, and sports equipment, where both mechanical strength and lightweight materials are required.^3–5^ Despite these advantages, conventional CFRPs are generally based on thermoset resin matrices, which have excellent thermal and chemical stability but are inherently brittle and irreversibly crosslinked, and thus, are hard to recycle and reuse. Additionally, such thermoset-based CFRPs are often disposed of by land filling or incineration towards the end of their life cycle, causing environmental and human health concerns.^6,7^ Therefore, one cost-effective solution to these problems is to manufacture thermosetting resins from reprocessable and bio-based raw materials to yield recyclable and degradable composites.

In recent years, polymeric matrices of covalent adaptable networks (CANs), especially vitrimers, have been of great interest for CFRPs because they have both the excellent mechanical performances of thermosets and the processability and recyclability of thermoplastics.^8–10^ Vitrimers are a class of CANs characterized by their associative mechanism of dynamic covalent bond exchanges. These associative exchanges in vitrimers result in a fixed crosslink density during bond exchange and thus exhibit minimal changes in their macromolecular structure.^11–14^ The concept of vitrimers was first introduced by Leibler and co-workers in 2011, who explored a transesterification mechanism in an epoxy network exhibiting flow and reprocessability at elevated temperatures.^15,16^ Since then, vitrimer materials have been used to create sustainable functional materials in many different fields, such as adhesives,^17^ coatings,^18,19^ composites,^20–23^ 3D printing,^24–26^ flexible electronics,^27^ and electromagnetic interference (EMI) shielding materials.^28^ Their stress relaxation, weldability, and self-healing behavior have made these materials a sustainable alternative to traditional thermosets. For aerospace and wind energy applications, the vitrimer materials are promising matrices for flexible composite parts. For example, strong and flexible materials are required for the fabrication of structural components, such as aircraft stringers or wind turbine parts, to withstand constant mechanical stress, wind loads, or vibrations, while also being lightweight and resistant to damage.^29,30^

Apart from the first introduced transesterification mechanism, various other dynamic covalent chemistries have been explored in vitrimer systems. Some of these, such as imine exchange,^31,32^ disulfide exchange,^22,33^ Diels–Alder exchange,^34^ acylsemicarbazide bonds,^35,36^ and diselenide bond exchange^37^ have also been effectively incorporated into vitrimer-based CFRPs to improve their adaptability, performance, and value for a wide range of structural applications. Degradable and recyclable resin matrices for CFRPs can also be readily prepared by introducing such dynamic exchangeable bonds. Yue *et al.*^38^ synthesized a recyclable, bio-derived epoxy resin based on imine bonds, which provided improved mechanical properties and acid-responsive degradability. The resin allows complete recovery and reuse of carbon fibers from CFRPs, promoting sustainable composite development. In another study by Si *et al.*,^29^ high-performance CFRPs based on epoxy vitrimer were developed, which enabled catalyst-free stress relaxation and effective degradation through thiol-mediated disulfide exchange, and thus make recovery and reuse of carbon fibers possible without losing their mechanical performance. While traditional vitrimers depend on a single type of exchangeable bond, recent developments have introduced dual dynamic bond-based vitrimers, which combine two different reversible chemistries in one network. This dual dynamic bond approach promotes stress relaxation, faster healing, and greater mechanical stability, enabling reprocessability and recyclability under varied conditions.^39^ When applied to CFRPs and adhesive materials by Verdugo *et al.*,^40^ the dual dynamic imine and disulfide bonds in a vitrimer matrix enabled a catalyst-free bond exchange, allowing the network to exhibit low creep and rapid stress relaxation. A bio-based vitrimer was also developed by Xu *et al.*,^41^ which combined dual dynamic hydroxyl ester and disulfide bonds into the CFRP matrix, where chemical degradation in three different solvents demonstrates the recyclability of these materials, which facilitates a complete recovery of undamaged carbon fibers.

In the current work, we prepare carbon fiber-reinforced composites consisting of a conventional epoxy and a soybean oil-based acrylic bio-precursor for the development of a dual dynamic vitrimeric matrix. Acrylic matrices in CFRPs offer superior long-term durability under environmental stress, particularly in applications exposed to prolonged UV radiation and outdoor conditions. Several studies have shown that while traditional epoxy matrices may provide high initial mechanical strength, acrylic-based systems exhibit enhanced environmental resistance and aging stability, and improve interfacial adhesion between carbon fibers.^42–44^ This makes them particularly well-suited for advanced composite applications in outdoor infrastructure, aerospace, and wind energy systems, where long-term durability is critical. The incorporation of a bio-based precursor offers multiple advantages as it provides a sustainable approach to prepare the CFRP matrix, while also improving the recyclability and degradability of the resulting network.^45^ To prepare the matrix, the DGEBA-based epoxy and the acrylated epoxidized soybean oil (AESO) precursor were cured with DTBA hardener, and tin(ii) 2-ethylhexanoate (Sn(Oct)_2_) was added as a transesterification catalyst. The AESO consists of long aliphatic chains in its structure, which provides flexibility to the network, while the rigid aromatic rings of the epoxy provide the mechanical rigidity. The prepared matrix shows vitrimeric behaviour such as stress relaxation and self-healing, attributed to the incorporation of dynamic transesterification and disulfide bond exchanges. The CFRPs were prepared using a vacuum-assisted resin infusion molding (VARIM) process for the fabrication of flexible composites, and their dynamic, mechanical, and recycling performance were evaluated.

## Experimental section

### Materials

Diglycidyl ether of bisphenol-A (DGEBA) resin (340.41 g mol^−1^), 2,2′-dithiodibenzoic acid (DTBA) hardener (306.36 g mol^−1^), imidazole (IM) initiator (68.08 g mol^−1^), tin(ii) 2-ethylhexanoate, ethylene glycol (EG), acrylic acid, triphenylphosphine (TPP), and dimethylformamide (DMF) were obtained from Sigma-Aldrich. Butylated hydroxytoluene was purchased from Thermo Fisher. All products were commercially available and used without requiring further purification. The epoxidized soybean oil was procured from Haryana Enterprises (KLJ Group), India. A unidirectional CF cloth (3 K, 200 g m^−2^) was obtained from Composite Tomorrow, India.

### Synthesis of acrylated oil from epoxidized soybean oil

Acrylated epoxidized soybean oil (AESO) was synthesized by reacting 30.0 g of epoxidized soybean oil with 4.58 g of acrylic acid in a 250 mL round-bottom flask, which was equipped with a reflux condenser, thermometer, and nitrogen atmosphere. To suppress unwanted side reactions, 0.027 g of butylated hydroxytoluene was added, which is a free radical inhibitor, followed by 0.3 g of triphenylphosphine to catalyze the reaction.^46^ The reaction was continued overnight at 65–75 °C. The reaction mixture was brought to room temperature, diluted with diethyl ether, and purified by washing with deionized water till it reached neutral pH.

### Preparation of bio-based epoxy vitrimers

Epoxy vitrimers were synthesized by physically mixing DGEBA and AESO in specified weight ratios (as listed in Table 1). Initially, the calculated amounts of DGEBA and AESO were added to a beaker and heated to 80 °C under continuous magnetic stirring to ensure uniform mixing and homogeneity, as both components have different viscosities and chemical structures. At this elevated temperature, AESO a viscous liquid disperses effectively within the DGEBA matrix, forming a single-phase mixture. Once a uniform blend was achieved, 1 wt% imidazole was added to initiate epoxy ring-opening, followed by 2 wt% DTBA as the curing agent. After ensuring complete dissolution of DTBA, (Sn(Oct)_2_) was introduced to catalyze transesterification. To ensure consistency in catalyst concentration across all formulations, a mass ratio approach was adopted instead of using mole ratios to maintain a uniform loading of the transesterification catalyst in each formulation. Utilizing mole ratios would have led to variations in the total weight of the individual components, thereby altering the catalyst concentration and potentially affecting the rate of transesterification. Such inconsistencies could result in variability in the dynamic behavior of the vitrimers, making it difficult to compare their performance reliably. This process ensured that both DGEBA and AESO were thoroughly incorporated into the reactive epoxy vitrimer network, allowing the formation of dynamic covalent bonds and a homogeneous cured matrix. The process begins with a nucleophilic attack by imidazole on the highly strained epoxide ring, leading to ring opening. The resulting intermediate interacts with a carboxylic acid (–COOH) group present in the system, abstracting a proton and generating a carboxylate ion. This stabilized intermediate can further react with another epoxide moiety. The carboxylate anion undergoes a nucleophilic reaction with an epoxide group, forming ester linkages. This reaction propagates, resulting in an extensively crosslinked polymer network in which multiple epoxide groups are converted into ester crosslinks.

**Table 1:** Different proportions of epoxies, initiator, hardener and catalyst to prepare epoxy vitrimers

S. no.	Sample code	Ratio of DGEBA/AESO/DTBA/Sn(Oct)_2_	DGEBA	AESO	Initiator (1 wt%)	Hardener (2 wt%)	Sn(Oct)_2_ (10 wt%)
1	D100B0	100/0/2 wt%/10 wt%	2 g	0 g	20 mg	40 mg	200 mg
2	D70B30	70/30/2 wt%/10 wt%	1.4 g	0.6 g	20 mg	40 mg	200 mg
3	D50B50	50/50/2 wt%/10 wt%	1 g	1 g	20 mg	40 mg	200 mg
4	D30B70	30/70/2 wt%/10 wt%	0.6 g	1.4 g	20 mg	40 mg	200 mg
5	D0B100	0/100/2 wt%/10 wt%	0 g	2 g	20 mg	40 mg	200 mg

**Scheme 1:**
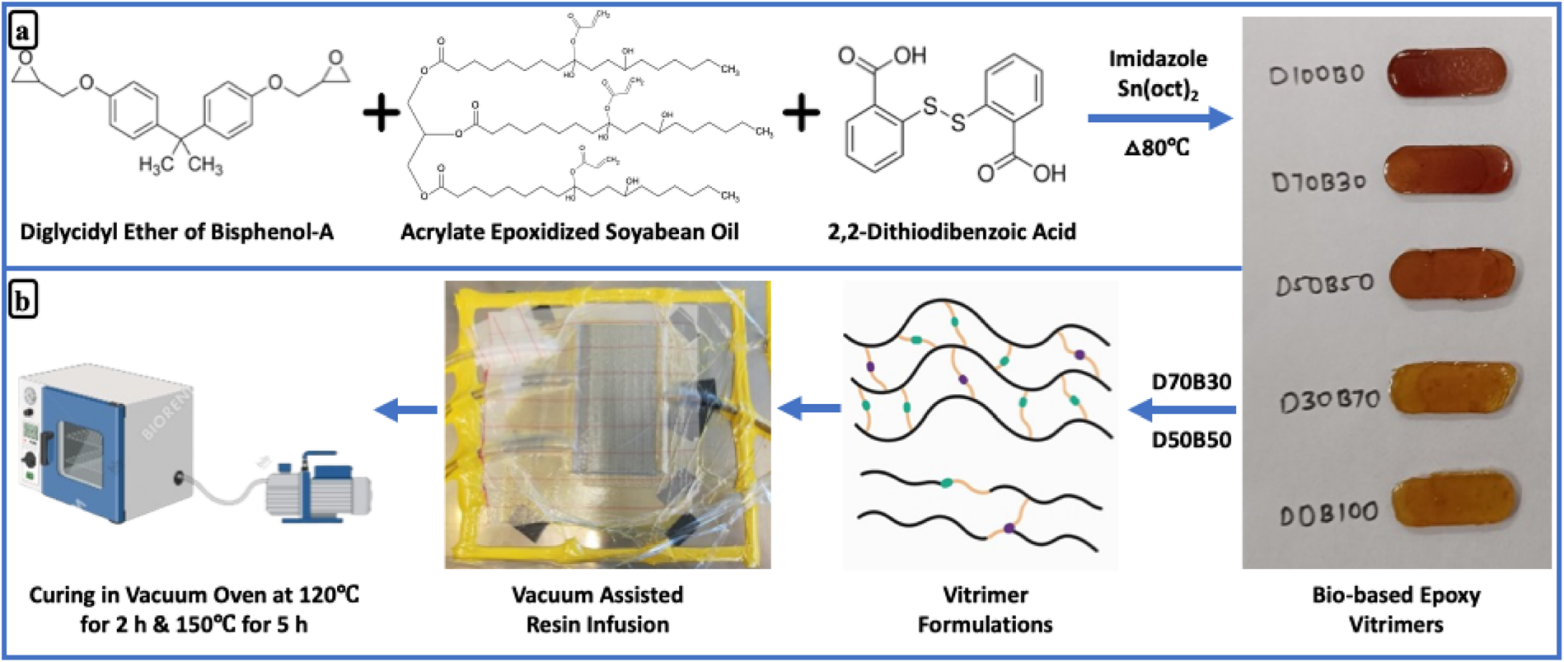
(a) Preparation of bio-based epoxy vitrimers; (b) preparation of carbon fiber vitrimer composites.

The resultant viscous formulation was poured into molds and cured in a staged manner: 80 °C for 2 hours, 120 °C for 2 hours, and finally 150 °C for 5 hours. This curing sequence enabled the formation of a robust, reprocessable vitrimer network, characterized by reversible covalent bonding facilitated through both transesterification and disulfide exchange mechanisms (Scheme 1a). To address the concern regarding the low DTBA concentration for vitrimer formation, epoxy thermosets with higher DTBA concentrations (5, 10, and 15 wt%) were also prepared using the same method, as illustrated in Fig. S1. Subsequently, the solubility analysis of these samples was conducted, as shown in Fig. S2.

### Preparation of carbon fiber vitrimer composites

Carbon fiber vitrimer composites were fabricated using a vacuum-assisted resin infusion molding system. Four layers of unidirectional carbon fibers, each cut to dimensions of 11 cm × 11 cm × 0.1 mm, were sequentially arranged on a steel plate and covered with peel ply, infusion mesh, and vacuum bagging film. Simultaneously, D70B30 and D50B50 bio-based epoxy vitrimers solutions were prepared by mixing at 80 °C. After thorough mixing, the solution was degassed and infused into the vacuum bag through a PVC hose, ensuring complete saturation of the carbon fibers. The infused composite plate was then pre-cured in a vacuum oven at 120 °C for 2 hours, followed by post-curing at 150 °C for 5 hours, as illustrated in Scheme 1(b).

### Characterization

Fourier Transform Infrared (FTIR) spectra were collected on a PerkinElmer spectrophotometer using attenuated total reflection (ATR) mode. Data acquisition involved 16 scans from 4000 to 400 cm^−1^ at a resolution of 4.0 cm^−1^. The glass transition temperature (*T*_g_) of the cured samples was determined using differential scanning calorimetry (DSC) on a Hitachi High-Tech Science DSC7020 instrument. Approximately 8–10 mg of each sample was placed in a pierced aluminium pan and analyzed under a N_2_ flow of 50 mL min^−1^. The thermal stability of the cured samples was investigated using thermogravimetric analysis (TGA) on a Hitachi High-Tech Science STA200 instrument. Approximately 10 mg of each sample was subjected to thermal degradation from 30 °C to 800 °C at a heating rate of 10 °C min^−1^ under a continuous flow of N_2_ at 100 mL min^−1^. To assess the material properties, the gel content of the samples was determined. Dry samples (approximately 30 mg, initial weight *W*_0_) were extracted in 5 mL of acetone for 24 hours. Following extraction, the samples were dried in an oven at 40 °C for 5 hours and reweighed (*W*_1_). The gel content was calculated using the following eqn (1):1
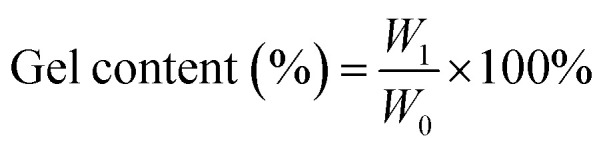


Thermomechanical properties were measured using a TA instruments TMA-Q400 equipped with a three-point bending clamp. Rectangular samples of about 15 mm × 5 mm × 0.5 mm were analyzed at 0.50 Hz, 0.1% strain, and from 40 to 120 °C at 3 °C min^−1^. Stress–strain experiments were conducted using a strain ramp mode with a constant force of 0.02 N. Strain measurements were performed at a constant temperature of 40 °C. Stress relaxation tests were conducted by first equilibrating samples at temperatures around the glass transition temperature (*T*_g_). A constant strain of 1% (within the linear viscoelastic region) was then applied to the sample, and the subsequent stress decay was monitored as a function of time. The self-healing capability of the cured sample was assessed by introducing a scratch using a razor blade. Subsequently, the samples were subjected to thermal treatment in an oven at 80 °C for durations of 50 and 60 min. The healing progress of the scratch was then observed using an Olympus BX51 optical microscope. The healing efficiency is considered to be the ratio of the healed scratch width to the original scratch width of the samples, which is calculated by the following eqn (2):2
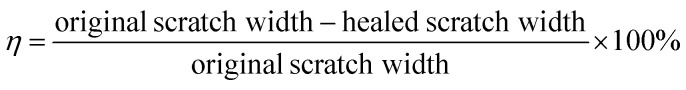
where *η* represents the healing efficiency.

To assess the solvent resistance behavior of the epoxy vitrimers, the samples (10 × 10 × 1.5 mm^3^) were submerged in tetrahydrofuran, acetone, ethanol, toluene, NaOH, and HCl for 72 hours. Additionally, the degradability of the epoxy vitrimers was evaluated by dissolving the samples in DMF at 70 °C for 4 hours.

Further, rectangular specimens of carbon fiber vitrimer composites, with dimensions of 100 mm × 10 mm × 2 mm, underwent tensile strength testing using a universal testing machine. The tests were performed at a constant temperature of 26 °C and a strain rate of 2 mm min^−1^. To ensure data reliability, each composition was tested three times. The flexural strength (*σ*_f_) and flexural modulus (*E*_f_) and strain at failure (*ε*_f_) on the carbon fiber vitrimer composites can be estimated by:3
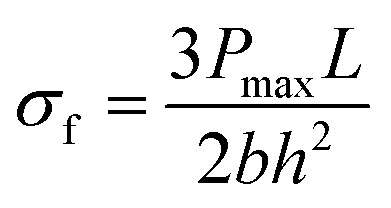
4
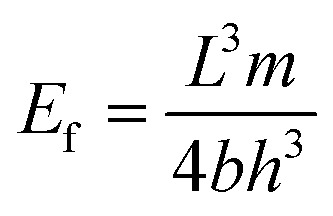
5
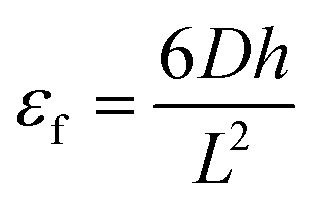
where *D* is the deflection of the composites. We selected a span to thickness ratio for the composites of *L*/*h* = 32. The span, width, and thickness of the flexural specimens were around 48, 10 and 1.5 mm, respectively.

To investigate the degradation process of the D70B30 composite and the recovery of carbon fibers, the composite samples were treated in DMF/EG at 70 °C for 4 hours. The degradation products were characterized through FTIR spectroscopy. The structural analysis of both the original and recovered carbon fibers was performed using X-ray diffraction (XRD) with a D8 ADVANCE ECO-Bruker system, operating at 40 kV, scanning over a 2*θ* range of 0.5 to 80°, and maintaining a speed of 5° min^−1^. Additionally, the surface morphology of virgin and recycled carbon fibers was analyzed with a ZEISS scanning electron microscope (SEM) under an accelerating voltage of 20 kV.

## Results and discussion

### Fundamental properties of bio-based epoxy vitrimers

**Fig. 1:**
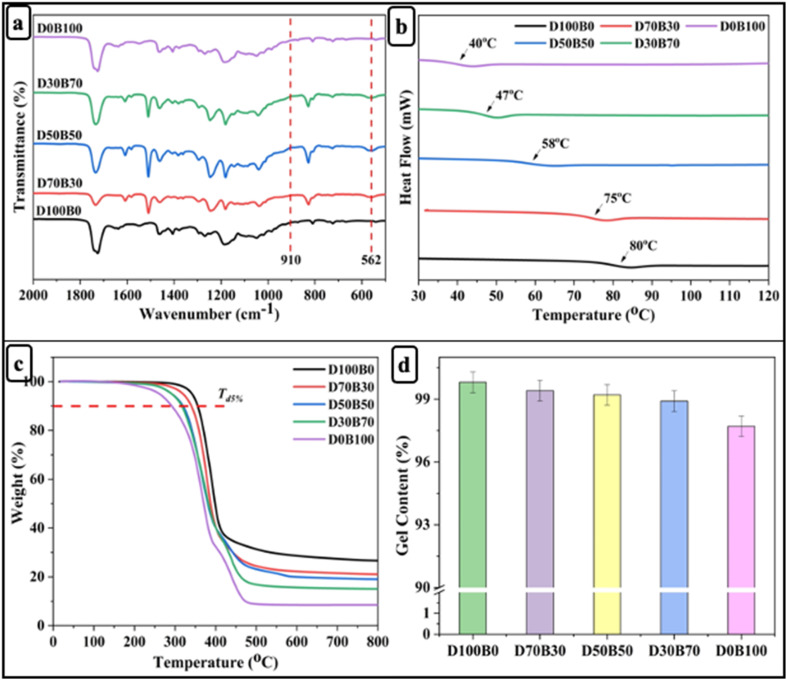
(a) FT-IR spectra; (b) DSC thermograms; (c) TGA curves; (d) gel content of bio-based epoxy vitrimers.

The formation of AESO was validated through ^1^H NMR analysis using deuterated chloroform (CDCl_3_) as the solvent (Fig. S3 and S4). The protons from methylene groups positioned between two carbon–carbon double bonds in the fatty acid chain appeared in the range of 2.0–2.5 ppm (H_f_). Signals at approximately 5.76, 6.29–6.34, and 6.02–6.06 ppm were attributed to the vinyl protons of the acrylate moiety (CH_2_=CH–), specifically corresponding to H_c_, H_a_, and the methyl group proton H_b_ in the CH_2_–CH segment. Additionally, the disappearance of the characteristic epoxy signal near 3 ppm in the modified AESO confirms successful acrylation.^47^ The AESO was synthesized by reacting epoxidized soybean oil with acrylic acid, and the formation of AESO was confirmed using FTIR spectroscopy (Fig. S5). The peak of the epoxy doublet disappears from AESO, which can initially be seen in the FTIR graph of epoxidized oil. The characteristic peaks of acrylate groups in AESO can be observed at 1635 cm^−1^ (acrylic acid double bond), and at 986 cm^−1^ a vinyl peak is also observed, further confirming acrylate groups in the AESO.^47^ The fabrication of cross-linked networks with dynamic properties was carried out following the methods outlined in previous studies.^22^Fig. 1(a) presents the FTIR spectra of bio-based epoxy vitrimers. The disappearance of the characteristic epoxy absorption peak at 910 cm^−1^ confirms the complete consumption of epoxy groups during the curing process. Additionally, the S–S stretching vibration at 562 cm^−1^, associated with disulfide bonds, is evident in all the spectra, indicating the successful incorporation of disulfide groups into the epoxy vitrimers.^48^ Moreover, the peak around ∼1750 cm^−1^ corresponds to the C=O stretching of ester groups, which increases with higher AESO content due to its acrylated structure rich in ester functionalities. The ∼1520 cm^−1^ peak is associated with aromatic ring vibrations and N–H bending from imidazole or DTBA, more prominent in DGEBA-rich systems due to the aromatic backbone and potential interactions with the curing agents. The 1200–1300 cm^−1^ region includes contributions from C–O–C and C–O stretching vibrations in ester and ether linkages, which vary depending on the degree of epoxy ring opening and the relative abundance of aliphatic AESO *versus* aromatic DGEBA. These spectral shifts and intensity changes thus reflect the structural evolution and composition-dependent crosslinking in the vitrimer network.

The curing behavior of epoxy mixtures was investigated using DSC. D100B0, D70B30, D50B50, D30B70, and D0B100 samples displayed baseline shift at 80 °C, 75 °C, 58 °C, 47 °C, and 40 °C, respectively (Fig. 1(b)), confirming the occurrence of curing reactions between epoxy and carboxyl groups within the feedstock. A three-stage curing protocol was employed: 80 °C for 2 hours, followed by 120 °C for 2 hours, and finally 150 °C for 5 hours. This multi-step process was designed to ensure complete curing while minimizing the risk of material degradation.^18^

The thermal stability of epoxy vitrimer networks with varying DGEBA/AESO ratios was investigated using TGA, as illustrated in Fig. 1(c). All samples displayed high thermal stability up to approximately 300 °C, with negligible weight loss in this region, indicating effective crosslinking and minimal presence of volatile compounds. The degradation temperature at 5% weight loss (*T*_d5%_) served as a reference point to compare the thermal robustness of each formulation. As expected, the D100B0 formulation (100% DGEBA) showed the highest *T*_d5%_, owing to its rigid aromatic backbone and dense epoxy crosslinking.^22^ In contrast, the fully bio-based D0B100 formulation exhibited the lowest thermal stability, attributed to the presence of flexible aliphatic chains and thermally labile ester groups in AESO.^49^ Interestingly, the intermediate formulations, D70B30 and D50B50, demonstrate a favorable balance between thermal performance and bio-based content. These samples retained relatively high *T*_d5%_ values while also incorporating significant amounts of AESO, offering improved material flexibility along with enhanced sustainability.

The gel content test provides valuable insights into the extent of cross-linking within the network.^50^ As illustrated in Fig. 1(d), the D70B30 and D50B50 bio-based epoxy vitrimers exhibit gel content values comparable to the D100B0 sample. These results are consistent with the thermal stability trends observed in TGA, suggesting an optimal balance between material stability and flexibility.

### Dynamic properties of bio-based epoxy vitrimers

**Fig. 2:**
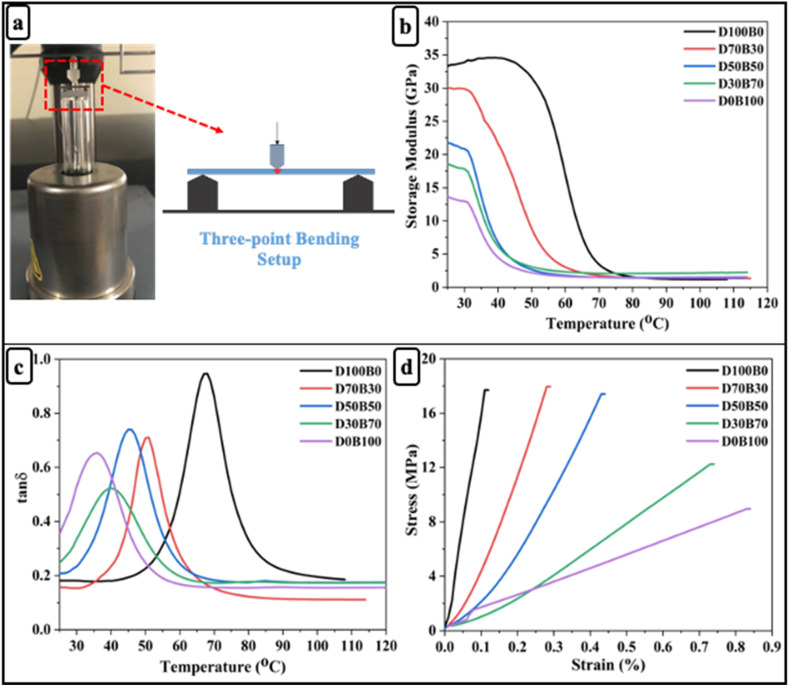
(a) Three-point bending setup; (b) storage modulus; (c) tan *δ*; (d) stress–strain relationship of bio-based epoxy vitrimers.

Three-point bending tests were conducted to investigate the mechanical response of the epoxy vitrimers under flexural loading (Fig. 2(a)). Fig. 2(b) illustrates the storage modulus *vs.* temperature curves for the bio-based epoxy vitrimer samples. At 25 °C, the D100B0 sample (33 GPa) exhibited the highest initial storage modulus, indicative of superior stiffness and a highly rigid aromatic structure.^51^ With increasing AESO content, a progressive decrease in storage modulus was observed, reflecting enhanced chain mobility due to the flexible aliphatic segments. However, the D70B30 (30 GPa) and D50B50 (21 GPa) samples retained relatively high storage modulus values compared to other AESO-rich formulations, suggesting an optimal balance between mechanical stiffness and the flexibility imparted by the bio-based component. According to Flory's ideal rubber elasticity theory, the crosslink density (*ν*) of the vitrimer network was determined using the storage modulus (*E*′) obtained from dynamic mechanical analysis. Specifically, the value of *E*′ at *T*_g_ + 50 °C was used, as this temperature ensures the material is in the rubbery plateau region, where elasticity dominates and entanglement effects are minimized. The crosslink density was calculated using the equation:6
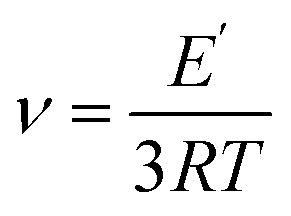
where *E*′ is the storage modulus at *T*_g_ + 50 °C, *R* is the universal gas constant (8.314 J mol^−1^ K^−1^), *T* is the absolute temperature (in Kelvin) at which *E*′ is measured.

Fig. 2(c) presents the tan *δ vs.* temperature curves for epoxy vitrimer formulations with varying DGEBA/AESO ratios and tabulated in Table 2. The peak of each curve corresponds to the *T*_g_, indicating the transition from a glassy to a rubbery state.^52^ The D100B0 sample exhibited the highest *T*_g_ (∼67 °C), reflecting a densely crosslinked and rigid network resulting from the aromatic backbone of DGEBA. With increasing AESO content, the *T*_g_ values progressively decreased (D70B30 (∼50 °C), D50B50 (∼45 °C), D30B70 (∼40 °C), and D0B100 (∼36 °C)) due to the incorporation of flexible aliphatic chains. Notably, the D70B30 and D50B50 samples demonstrate a favorable balance between rigidity and molecular mobility. Their *T*_g_ values remained sufficiently high to ensure structural stability, while the broader tan *δ* peaks suggest enhancement in molecular mobility and potentially a broader range of damping capabilities.^53^

**Table 2:** The detailed results of thermal, dynamic and self-healing properties of samples

Sample name	*T*_g_ (°C)		Flexural strength (MPa)	Flexural modulus (GPa)	Storage modulus (GPa)	*T*_d5%_ (°C)	Gel content (%)	Self-healing efficiency (%)	*ν* (10^−3^ mol cm^−3^)
	TMA	DSC							
D100B0	67	80	17.8	34.7	33	358	99.8	88	1.24
D70B30	50	75	17.9	32.3	30	342	99.4	92	1.21
D50B50	45	58	17.5	31.6	21	325	99.2	90	1.19
D30B70	40	47	12.5	26.7	18	318	98.9	88	1.05
D0B100	36	40	8.9	19.4	13	294	97.7	87	0.93

The stress–strain relationship of each formulations is depicted in Fig. 2(d). The D100B0 sample demonstrated the highest stiffness and elastic modulus, indicated by the steepest slope and lowest strain at break (∼0.12%). With the incorporation of AESO, a noticeable decrease in stiffness was observed, accompanied by an increase in strain. Notably, D70B30 and D50B50 samples maintained considerable flexural strength (∼17.5 MPa) while showing improved strain values (∼0.27–0.43%), suggesting a desirable balance between strength and ductility. In contrast, the D30B70 and D0B100 samples exhibited lower flexural strength but significantly higher failure strains, indicating enhancement in flexibility due to the aliphatic nature of AESO.

### Stress relaxation of bio-based epoxy vitrimers

**Fig. 3:**
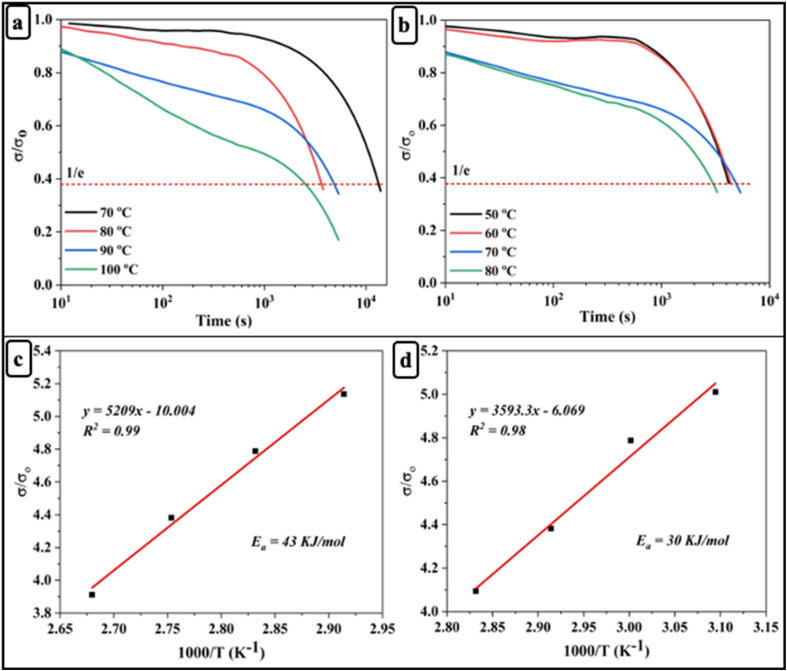
(a) Stress-relaxation for D70B30; (b) stress-relaxation for D50B50 at different temperatures; (c) ln(*τ*) *versus* 1000/*T* plot for D70B30; (d) ln(*τ*) *versus* 1000/*T* plot for D50B50 bio-based epoxy vitrimers.

Stress relaxation tests were conducted to investigate the topological rearrangement behavior of epoxy vitrimers incorporating dynamic disulfide and hydroxyl–ester bonds. The relaxation time (*τ*), defined as the time required for the stress or modulus to decay to 1/*e* (36.7%) of its initial value.^54^ The temperature dependence of *τ* for D70B30 and D50B50 is illustrated in Fig. 3(a) and (b). Increasing the temperature from 70 °C to 100 °C for D70B30, and from 50 °C to 80 °C for D50B50, resulted in a decrease in *τ* from 170 min to 50 min, and from 150 min to 60 min, respectively. This reduction is attributed to the accelerated exchange rate of dynamic disulfide and hydroxyl–ester bonds at elevated temperatures, which, combined with enhanced chain segment mobility, facilitates network rearrangement.^55^ The relaxation time of bio-based epoxy vitrimers exhibits Arrhenius-type behavior, as described by the following eqn (3):^56^7
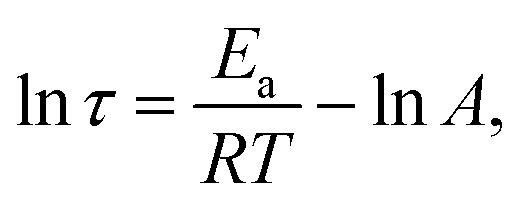
where *τ* is the relaxation time, *T* is the absolute temperature, *E*_a_ is the activation energy for the dynamic bond exchange reaction, *R* is the universal gas constant, and *A* is the pre-exponential factor. As shown in Fig. 3(c) and (d), the linear fit to the data for D70B30 and D50B50 yielded activation energies (*E*_a_) of 43 kJ mol^−1^ and 30 kJ mol^−1^, respectively. These values are consistent with previously reported values for vitrimer networks based on dual-dynamic bond exchange reactions.^57^ Notably, the relaxation curve of D70B30 at 80 °C shows a more rapid decay compared to other temperatures, and the curves for D50B50 at 50 °C and 60 °C are nearly overlapping. These deviations from monotonic relaxation behavior can be attributed to the transition zone around *T*_g_, where a small increase in temperature leads to a sudden rise in segmental mobility and dynamic bond exchange. In D70B30 (*T*_g_ ∼75 °C), testing at 80 °C pushes the network into the rubbery regime, enabling faster rearrangement. In D50B50 (*T*_g_ ∼58 °C), 50 °C and 60 °C straddle the glass transition, where dual dynamic exchanges and local heterogeneities may result in comparable relaxation rates. Such nonlinearities are common in associative vitrimer systems, as observed by Rohewal and co-workers.^58^

### Self-healing, solvent resistance and degradability behaviour of bio-based epoxy vitrimers

**Fig. 4:**
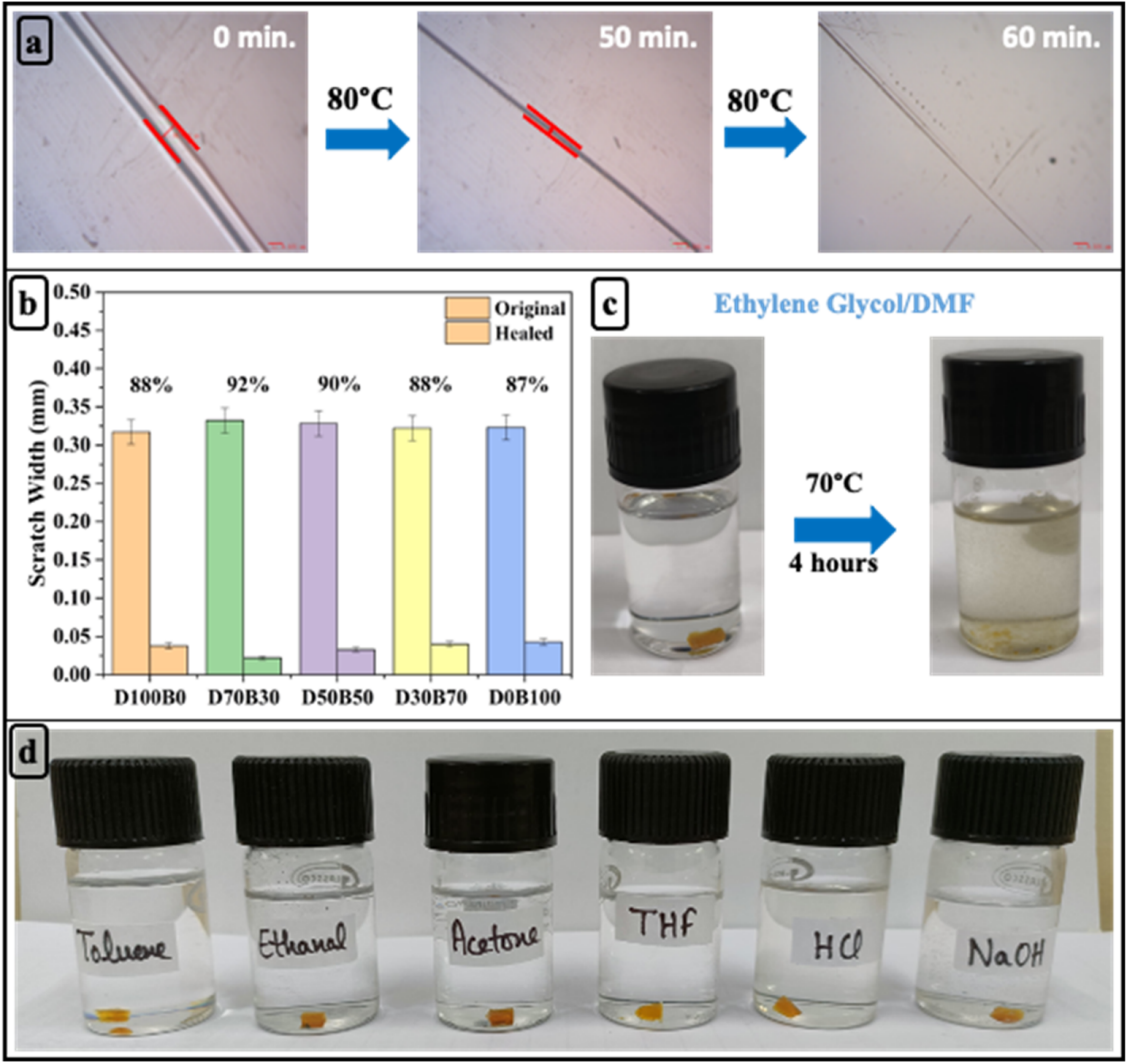
(a) Self-healing images of D70B30; (b) healing efficiency of bio-based epoxy vitrimers; (c) degradation of D70B30; (d) solvent resistance of bio-based epoxy vitrimers.

The self-healing behavior of the bio-based epoxy vitrimer samples was evaluated based on dual dynamic exchange reactions activated by thermal stimulus. Optical microscopy images were used to monitor the healing of surface cracks at 80 °C over 50 and 60 minutes (Fig. 4(a)). Initially, the scratches on the surface were clearly visible. After 50 minutes, the cracks visibly diminished, and by 60 minutes, the traces were barely noticeable, indicating progressive healing. To further validate the self-healing performance, the healing efficiency of the different samples was quantified and presented in the Fig. S6 using the method described in the characterization section. As shown in Fig. 4(b), the results revealed that the D70B30 (∼92%) and D50B50 (∼90%) samples exhibited significantly higher healing efficiencies compared to the other formulations. This enhancement in performance is attributed to the synergistic effect of dual dynamic bonds and increased segmental mobility introduced by the bio-based AESO, which together promote efficient bond exchange and network rearrangement for effective crack closure.^59^

The degradability of the bio-based epoxy vitrimers was evaluated by immersing the samples in DMF and EG (1 : 1 mixture) at 70 °C for 4 hours. As time progressed, the solution became darker, and the bio-based epoxy vitrimer began to dissolve. This dissolution is likely due to the breakdown of the dynamic covalent disulfide bonds and the hydroxyl–ester groups under the harsh conditions, which leads to the weakening of the polymer network, making it more susceptible to solvent attack.^60^Fig. 4(c) demonstrates the degradation of the D70B30 sample, visually highlighting the material's response to the degradation process.

The solvent resistance of the bio-based epoxy vitrimers was evaluated by immersing the samples in various solvents. All samples demonstrated excellent solvent resistance, which can be attributed to the dense crosslinked network structure and the inherent chemical stability provided by the dynamic covalent bonds. These features restrict solvent penetration and inhibit polymer chain disentanglement, thereby maintaining structural integrity.^60^ Representative images of the D70B30 sample after 72 hours of solvent immersion are illustrated in Fig. 4(d), highlighting the material's stability and durability across chemically aggressive environments.

### Dynamic and mechanical properties of carbon fiber vitrimer composites

**Fig. 5:**
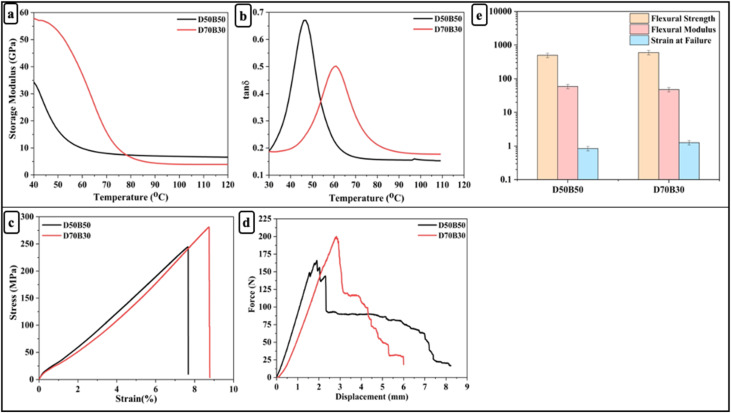
(a) Storage modulus; (b) tan *δ*; (c) stress *vs.* strain curves; (d) force *vs.* displacement curves; (e) flexural strength, modulus, and strain at failure of D50B50 and D70B30 bio-based epoxy vitrimers.

Based on their superior dynamic and mechanical behavior properties, D70B30-CF and D50B50-CF epoxy vitrimer were further studied to develop the fiber-reinforced composites (Table 3). As seen in Fig. 5(a) and (b), D70B30-CF demonstrates a higher storage modulus (58 GPa) and *T*_g_ (61 °C) compared to D50B50-CF (35 GPa and 47 °C, respectively), indicating enhanced crosslink density and thermal resistance. The tan *δ* peak is also broader and shifted to higher temperatures for D70B30-CF, reflecting better chain mobility at elevated temperatures while maintaining mechanical stability.

**Table 3:** The detailed results of dynamic and mechanical properties of samples

Sample name	Storage modulus (GPa)	*T*_g_ (tan *δ*)	Tensile strength (MPa)	Young's modulus (GPa)	Flexural strength (MPa)	Flexural modulus (GPa)	Strain at failure (%)
D70B30-CF	58	61	281	4.2	600	47.7	1.26
D50B50-CF	35	47	245	3.1	498	58.7	0.84

Mechanical characterization further supports the enhanced performance of D70B30-CF (Fig. 5(c) and (d)). Under tensile loading, D70B30-CF demonstrates a higher tensile strength of 281 MPa in comparison to 245 MPa for D50B50-CF, indicating superior load-bearing capacity. Flexural testing reveals that D70B30-CF exhibits a significantly higher flexural strength of 600 MPa, compared to 498 MPa for D50B50-CF, reflecting an improvement in resistance to bending-induced failure. While the flexural modulus of D70B30-CF (47.7 GPa) is slightly lower than that of D50B50-CF (58.7 GPa), this reduction in stiffness is offset by a substantial increase in flexibility, as evidenced by the higher strain at failure (Fig. 5(e)) (1.26% for D70B30-CF *vs.* 0.84% for D50B50-CF).^61^ This indicates that D70B30-CF not only withstands higher bending loads but also endures better deformation before failure, demonstrating a favourable balance between strength and ductility.

### Recycling of carbon fiber vitrimer composites

**Fig. 6:**
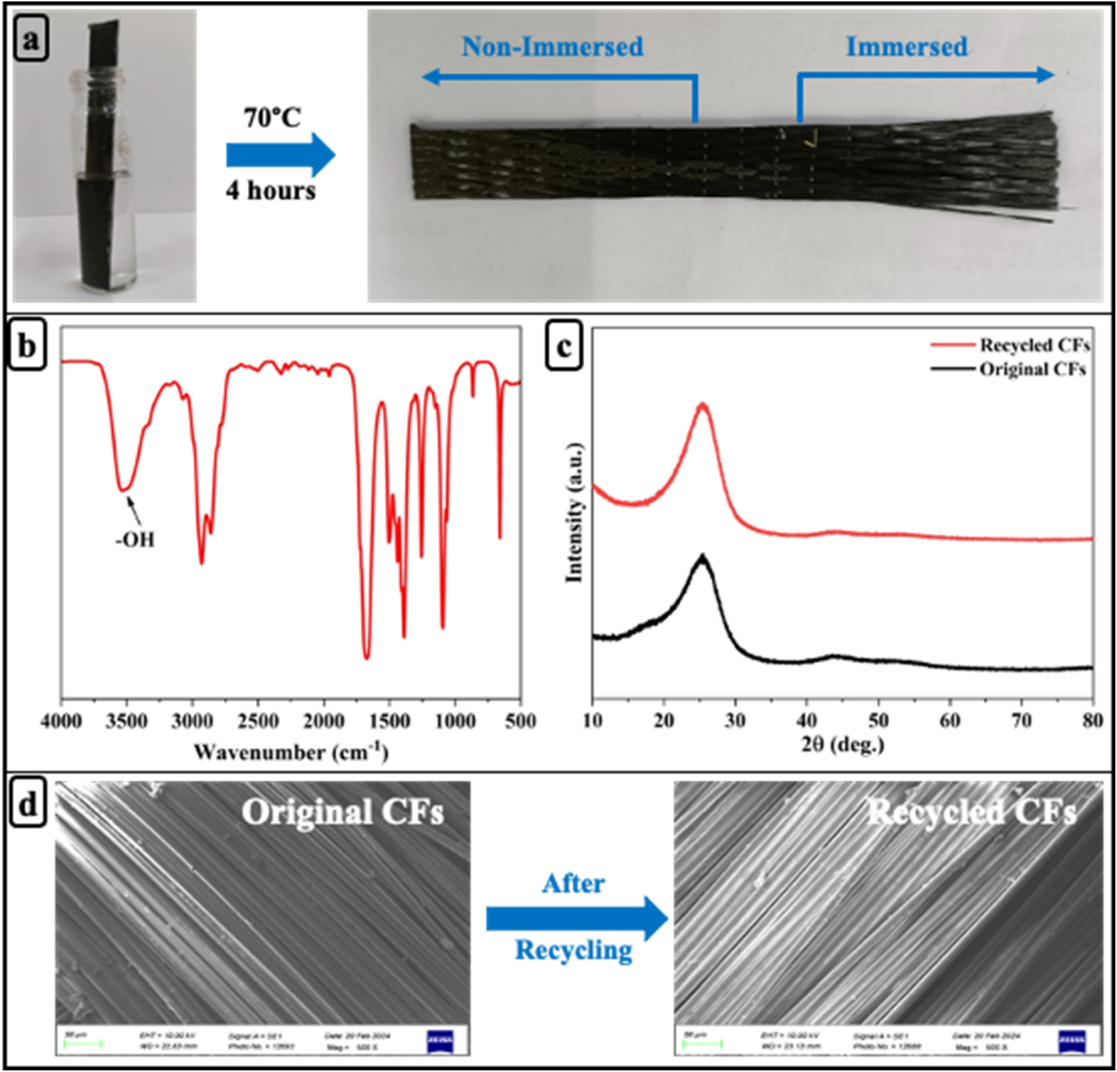
(a) Photographs of the recycling process of D70B30-CF vitrimer composite; (b) FT-IR spectra of D70B30-CF degraded product; (c) XRD; (d) SEM of original and recycled carbon fibers.

The vitrimer network incorporates dynamic disulfide and ester linkages, which undergo cleavage under thermal treatment in EG/DMF. While the polar aprotic DMF facilitates matrix swelling and bond destabilization, EG may contribute to ester bond cleavage *via* transesterification, ultimately leading to matrix degradation and fiber recovery. Based on this mechanism, EG/DMF was selected as the solvent system to degrade the D70B30 epoxy vitrimer matrix in D70B30-CF composites and recover the carbon fibers at 70 °C within 4 hours. As shown in Fig. 6(a), the composite undergoes visible degradation, and the carbon fibers are recovered undamaged after matrix breakdown. Fig. 6(b) displays the FTIR spectrum of the degraded product, highlighting a characteristic absorption peak at 3613 cm^−1^, attributed to hydroxyl functional groups. XRD analysis, shown in Fig. 6(c), reveals no significant structural differences between the original and recycled fibers, indicating that the chemical structure remained largely unaffected by the degradation process. Furthermore, SEM imaging in Fig. 6(d) confirms that the surface morphology of the recycled fibers remains smooth and comparable to that of the original carbon fibers.

## Conclusion

In this study, we successfully developed a dual dynamic bio-based epoxy vitrimer system using varying ratios of DGEBA and AESO. The addition of the bio-based AESO into the epoxy matrix contributed significantly to the flexural performance and flexibility of the final composites. The presence of long aliphatic chains in the AESO backbone imparts a plasticizing effect, which improves toughness and reduces brittleness, ultimately enhancing the material's ability to endure flexural stresses, as is also evident in the flexural studies. The incorporation of both transesterification and disulfide exchange mechanisms imparted the vitrimers with dynamic functionalities such as self-healing, stress relaxation, and reprocessability. Among the formulations, D70B30 and D50B50 exhibited an optimal combination of thermal stability, mechanical strength, and flexibility due to the synergistic effect of rigid aromatic and flexible aliphatic components. These vitrimer matrices were effectively used to fabricate high-performance CFRP laminates *via* VARIM, achieving excellent tensile and flexural properties. Furthermore, the vitrimer matrix enabled complete chemical degradation and recovery of intact carbon fibers under mild conditions, demonstrating a viable pathway for closed-loop recycling. Overall, this work presents a scalable and sustainable approach toward the design of recyclable CFRPs.

## Conflicts of interest

There are no conflicts to declare.

## Supplementary Material

NA-OLF-D5NA00624D-s001

## Data Availability

The data supporting this article have been included as part of the SI, and the file for the same has been uploaded with the submission. The supplementary information file contains supporting experimental data and characterization details relevant to the vitrimer formulations discussed in the main article. It includes a detailed rationale for using 2 wt% DTBA in DGEBA/AESO systems, synthesis and solubility analysis of epoxy vitrimers with higher DTBA concentrations. Additionally, it presents spectroscopic data for the synthesis of acrylated epoxidized soybean oil (AESO), including ^1^H NMR and FTIR spectra. Finally, the file provides the self-healing images of bio-based vitrimer formulations other than D70B30. See DOI: https://doi.org/10.1039/d5na00624d.
